# Does Knowledge of Clinical Case Outcome Influence Supervisor Evaluation of Resident Clinical Reasoning?

**DOI:** 10.1097/ACM.0000000000006122

**Published:** 2025-06-06

**Authors:** Charlotte van Sassen, Silvia Mamede, Walter van den Broek, Patrick Bindels, Laura Zwaan

**Affiliations:** **C. van Sassen** is a general practitioner, educator, and PhD candidate in medical education, Department of General Practice and Institute of Medical Education Research Rotterdam, Erasmus Medical Center, Rotterdam, the Netherlands; ORCID: https://orcid.org/0000-0003-0028-6689.; **S. Mamede** is associate professor, Institute of Medical Education Research Rotterdam, Erasmus Medical Center, and associate professor, Department of Psychology, Education, and Child Studies, Erasmus School of Social and Behavioral Sciences, Rotterdam, the Netherlands; ORCID: https://orcid.org/0000-0003-1187-2392.; **W. van den Broek** is professor in evidence based medical education and scientific director, Institute of Medical Education Research Rotterdam, Erasmus Medical Center, Rotterdam, the Netherlands; ORCID: https://orcid.org/0000-0003-3215-5640.; **P. Bindels** is professor in general practice and head, Department of General Practice, Erasmus Medical Center, Rotterdam, the Netherlands; ORCID: https://orcid.org/0000-0001-5941-4820.; **L. Zwaan** is associate professor, Institute of Medical Education Research Rotterdam, Erasmus Medical Center, Rotterdam, the Netherlands; ORCID: https://orcid.org/0000-0003-3940-1699.

## Abstract

**Purpose:**

This study examines whether outcome bias affects the assessment of general practice (GP) residents and explores supervisor feedback characteristics.

**Method:**

In a within-subjects experiment conducted in June 2023, Erasmus Medical Center GP supervisors reviewed 6 clinical vignettes with ambiguous diagnoses assessing residents’ diagnostic decisions. Each vignette had 2 versions, differing only in the final sentence indicating favorable or adverse clinical outcome. Supervisors were randomly assigned to review half the vignettes with favorable clinical outcomes and half with adverse clinical outcomes. Supervisors provided scores (range of 1–10, with 10 indicating exceptional achievement and 1–5 indicating insufficient performance) and feedback, analyzed for valence, content specificity, process versus outcome focus, and politeness strategies.

**Results:**

Sixty-two supervisors participated in the study. Vignettes ending in adverse clinical outcomes received lower scores versus those ending in favorable clinical outcomes (mean [SE] scores, 5.25 [0.12] vs 6.26 [0.16]; *P* < .001) and prompted more negative feedback (mean [SE] negative idea units, 2.35 [0.11] vs 1.80 [0.09]; *P* < .001). Negative feedback exhibited greater specificity than positive feedback (mean [SE] proportion of specific idea units, 0.88 [0.02] vs 0.44 [0.03]; *P* < .001), regardless of clinical outcome. Most feedback addressed process-related aspects (grand mean proportion of process-related idea units, 0.97; 95% CI, 0.95–0.98). Polite language was more prevalent in negative versus positive feedback (mean [SE] proportion of feedback with politeness strategies, 0.50 [0.04] vs 0.15 [0.02]; *P* < .001), regardless of clinical outcome.

**Conclusions:**

The study identified outcome bias in the evaluation of GP residents, with adverse clinical outcomes leading to lower scores and more negative, specific, process-focused feedback. Although such feedback can enhance learning, it may also hinder learning by triggering negative emotions. The findings emphasize the educational value of diagnostic errors but stress the need for objective assessment strategies to optimize learning opportunities.

Clinical reasoning (CR) is a cornerstone of a physician’s daily work and requires complex decision-making skills. Equipping medical students with strong CR skills is a critical facet of their education. The training of general practitioner (GP) residents usually includes educational sessions dedicated to enhancing their CR skills. Additionally, they gain practical experience in clinical settings where supervisors assess their CR skills. Although the goal is to assess residents as objectively as possible, retrospective assessments may be susceptible to hindsight and outcome bias (i.e., when knowledge of the result of a case may bias assessment).^1^ Hindsight bias refers to the tendency to overestimate one’s ability to predict the clinical outcome of a case after learning about it.^2^ Outcome bias, a related concept, occurs when the knowledge of the result of a case—whether favorable or adverse—unjustly influences judgments about the quality of the decision-making process that led to it.^3,4^ Knowing the result of a case can significantly affect the assessment of competence,^5^ and favorable outcomes tend to lead to more positive judgments.^1^

Outcome and hindsight bias have been observed in research in the medical context as well. A study by Zwaan et al^6^ revealed that the assessment of the diagnostic reasoning process in a clinical case by physicians was influenced by case outcome information. Particularly, in instances where an ambiguous case resulted in an incorrect diagnosis, twice as many errors were identified compared with cases in which the diagnosis was accurate, even though the diagnostic processes were identical in both scenarios. A study by Caplan^7^ showed that reviewers’ ratings of the appropriateness of care were inversely related to the severity of patient injury, with permanent impairment leading more frequently to judgments of inadequate care than temporary impairment.

Similarly, these biases may affect supervisors’ assessments of residents’ CR in practice, particularly in cases with an adverse clinical outcome due to a diagnostic error. Although diagnostic errors can provide valuable learning opportunities, for instance, by revealing knowledge gaps of more atypical disease presentations,^8,9^ they may affect supervisors’ evaluations. Knowledge that an error has occurred may lead to a tendency to shift the focus toward identifying and highlighting errors behind the clinical outcome, rather than discussing the complexity and nuances of the reasoning process. Moreover, having knowledge of the clinical outcome of a case might result in an undesired decrease in scores and an increase in the amount of (negative) feedback on the performance of the residents. The influence of feedback on learning and performance varies and is contingent on several factors, such as the timing of the feedback, the manner in which it is delivered by the feedback provider,^10^ the feedback environment (e.g., the safety of the environment), the interpretations of the recipient,^11^ the regulatory focus of the recipients,^12^ and the valence of the feedback (the intrinsic value or emotional tone of feedback, indicating whether it is positive or negative). Recipients of performance feedback, especially when it is negative, do not always accept it or make use of it. Positive evaluations tend to be better received and are perceived as more accurate than their negative counterparts,^13^ and recipients who concentrate on positive aspects of their performance, rather than the negative ones, are more likely to improve.^14^ Given that performance feedback relates to one’s personal abilities, it is often challenging to handle objectively and tends to be emotionally charged; negative feedback can harm the recipient’s self-esteem and pride,^15^ which can decrease performance^16^ and can have a lasting impact on subsequent behavior.^16–20^

A way to mitigate the adverse effects of increased negative feedback that is potentially triggered by knowledge of the clinical outcome of a case is to incorporate linguistic politeness strategies.^21,22^ These strategies include hedging (e.g., “could have,” “a little,” “fairly,” “as far as I can tell,” “I think”), impersonalization (omitting names or pronouns for distance), or conventional indirectness (e.g., “good,” “solid,” “met expectations”). Although the use of polite language in feedback may result in vagueness, it can soften criticism.^21^ It plays a crucial role in promoting smooth social functioning, and studies indicate better learning results compared with direct language.^23,24^

Given the established impact of outcome bias on judgments, possibly affecting the tone and focus of feedback and thereby influencing the learning process, it is essential to comprehend the effect of outcome bias on the evaluation of the CR skills of GP residents by their supervisors. Within clinical practice, diagnostic errors leading to adverse clinical outcomes may arise and are assessed by supervisors, offering valuable learning opportunities for residents if feedback is tailored to enhance learning and performance. This study explores differences in the feedback provided by supervisors in ambiguous clinical vignettes, each featuring 2 possible diagnoses and the same diagnostic process but with varying clinical outcomes (favorable vs adverse). In both versions, the resident made the same diagnostic error—failing to consider or test for the alternative diagnosis. However, in the favorable clinical outcome, this error had no consequences, and the patient recovered well, whereas in the adverse clinical outcome, it resulted in a missed diagnosis and patient harm. For example, a patient presenting with shortness of breath was treated with antibiotics based on the resident’s assumption of pneumonia, without considering or testing for a pulmonary embolism. In the favorable clinical outcome version, the patient recovered with antibiotics. In the adverse clinical outcome version, the patient deteriorated due to the undiagnosed pulmonary embolism.

This design allows us to examine whether and how knowledge of the clinical outcome influences the feedback supervisors provide. We assess the impact of clinical outcome knowledge by comparing diagnostic performance scores given by supervisors across case versions. Additionally, we evaluate the effect of supervisors’ own reflection on their feedback by comparing diagnostic performance scores given before and after the provide feedback. Furthermore, we analyze the quantity and characteristics of the feedback provided by supervisors by assessing its valence, specificity, and focus (process vs outcome oriented). We also examine the use of polite language in feedback. This analysis provides insight into the presence of outcome bias and how it manifests in the characteristics and quality of supervisors’ feedback on CR skills of residents. Understanding how outcome bias manifests within assessments and feedback is essential for identifying ways to mitigate this bias. By gaining this understanding, we can offer recommendations to supervisors on how to optimize their feedback in daily practice, ensuring it is as effective and beneficial as possible.

## Method

### Study participants and setting

Participants were supervisors of the GP vocational training at the Department of General Practice, Erasmus Medical Center, Rotterdam, the Netherlands. Written informed consent to participate in the study was obtained from participants. All methods were performed in accordance with relevant guidelines and regulations. Ethical approval was waived by the Medical Ethics Committee of Erasmus Medical Center Rotterdam because the rules laid down in the Medical Research Involving Human Subjects Act do not apply to this research.

The Erasmus Medical Center is the largest of the 8 academic hospitals in the Netherlands, with 1,215 beds.^25^ The Department of General Practice trains approximately 350 GP residents as part of a 3-year curriculum, which accounts for 14.7% of all GP trainees in the Netherlands, making it the largest GP training institution.^26^ During their postgraduate training at the Erasmus Medical Center, residents spend the first and third years working in various GP practices, whereas the second year is dedicated to diverse settings, such as hospital emergency departments, elderly care institutions, mental health facilities, or specialized hospital departments, such as pediatrics or dermatology. Residents practice 4 days a week under the supervision of a senior GP or medical specialist. Both residents and supervisors attend teaching sessions at the Department of General Practice, Erasmus Medical Center, 1 day per week and 1 day per month, respectively. CR is embedded in learning sessions during regular teaching sessions supervised by the departmental teaching staff as well as during daily clinical practice under one-on-one supervision by senior GPs. During the training sessions for supervisors, supervisors are taught how to deliver effective feedback to their residents across different settings, skill levels, and types of learning activities.

### Materials and procedure

#### Clinical vignettes development.

Six fictitious clinical vignettes with ambiguous diagnoses (2 probable diagnoses) were written by 2 GPs (a study investigator and an independent faculty member from the Department of General Practice). Two versions were developed for each vignette. Both versions were identical and written in the same structure, with the same clinical and patient information and the same diagnostic decisions of the resident. The only difference was in the last paragraph: in the version with a favorable clinical outcome, the patient improved with the prescribed treatment, meaning the resident’s hypothesized diagnosis was correct. In contrast, in the version with an adverse clinical outcome, the same diagnostic oversight had consequences—the patient did not improve with the same treatment, revealing that the resident’s hypothesized diagnosis was incorrect and the alternative diagnosis was missed. Crucially, the diagnostic processes in both versions were identical; in both cases, the resident failed to consider or test for the alternative diagnosis. This design highlights how identical diagnostic processes can be judged differently depending on the outcome, even when the underlying error remains unchanged (see Supplemental Digital Appendix 1 at http://links.lww.com/ACADMED/B732 for an example of a clinical vignette with an adverse or a favorable clinical outcome).

#### Procedure.

This within-subject experiment took place in June 2023, embedded in an educational program for supervisors during a 2-day educational seminar organized annually by the Department of General Practice, Erasmus Medical Center. During the session, participants received a link to a questionnaire (Qualtrics, Provo, Utah), a web-based survey tool, that presented them with the clinical vignettes. To prevent bias, participants were unaware of the purpose of the study during the session and debriefed after the session.

**Figure 1:**
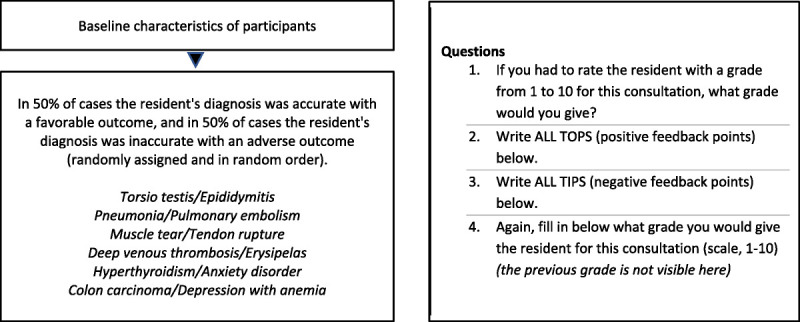
Study design overview and list of clinical vignettes for 62 general practice supervisors assessing residents’ diagnostic performance in 6 fictitious, ambiguous clinical case vignettes with favorable or adverse clinical outcomes, Erasmus Medical Center, June 2023.

Participants were randomly assigned to 1 of 6 variations of the questionnaire, which were created to counterbalance the vignette versions. In each variation, half of the vignettes had favorable clinical outcomes and the other half had adverse clinical outcomes, arranged in various combinations so that each participant viewed 3 vignettes with favorable clinical outcomes and 3 with adverse clinical outcomes. After completing informed consent and providing baseline characteristics, participants were exposed to the 6 ambiguous clinical vignettes in random order to control for any potential order effects. All participants were first asked to score the resident’s performance on a scale of 1 to 10 and then to provide free-text feedback in the form of TIPS (negative feedback points) and TOPS (positive feedback points). Finally, after delivering TIPS and TOPS feedback, participants were asked to rescore the residents on a scale of 1 to 10 without reference to their initial scores. This process aimed to determine whether scores changed after feedback, assessing the impact of reflection on scoring (see Figure 1 for study design overview).

In the Netherlands, students are typically graded on a scale of 1 to 10, with 6 being the minimum passing grade. This system is widely used across all levels of education and provides a clear, standardized method for evaluating academic performance, with 10 indicating exceptional achievement and 1 to 5 indicating insufficient performance. The TIPS and TOPS approach is commonly used in our GP education as a practical feedback method. This approach aligns with effective feedback practices that emphasize a balanced feedback method, highlighting strengths (TOPS) and identifying areas for improvement (TIPS).^10,16,27^

### Data analysis

The statistical analysis was performed with SPSS Statistics software for Windows, version 25 (IBM, Armonk, New York). Differences were considered significant at *P* < .05. An a priori power analysis was conducted using G*Power, version 3.1.9.6 (Heinrich Heine University, Düsseldorf, Germany)^28^ to determine the minimum sample size required to test the study hypothesis. Results indicated that the required sample size to achieve 80% power for detecting a medium effect at a significance criterion of *α* = .05 was 52 for a repeated-measures, within-factors analysis of variance (ANOVA).

#### Diagnostic performance scores.

First, mean diagnostic performance scores given to the residents in the 3 clinical vignettes with favorable clinical outcomes and in 3 vignettes with adverse clinical outcomes were calculated per participant, both before and after providing feedback. A repeated-measures ANOVA was conducted with vignette version (adverse vs favorable clinical outcome) and moment of scoring (before vs after providing feedback) as within-subjects factors to examine the effect of clinical outcome of the vignette and supervisors’ reflection on their own feedback on diagnostic performance scores.

#### Feedback coding.

To analyze the quantity and characteristics of the feedback, the free-text responses of TIPS and TOPS provided by supervisors were first divided into idea units by 2 senior GP staff members from the Department of General Practice (C.v.S., R.S.). An idea unit is the smallest meaningful idea that can be identified in a fragment of text.^29^ Given that content-specific feedback is considered more effective than generic feedback,^30^ the idea units were further coded as specific or generic. Feedback was scored as specific when it included detailed information about what went well or wrong, often with examples or suggestions. In contrast, generic feedback lacked specificity, using only vague statements, such as “good job,” “great,” or “not good.” Furthermore, to assess the assumption that feedback on known adverse clinical outcomes tends to focus more on the outcome rather than the process, the idea units were categorized into outcome- or process-focused items. We categorized feedback as related to clinical outcome when supervisors specifically addressed the result of the case or when their feedback clearly indicated that the outcome of the case was the primary focus. Conversely, feedback that did not reference the clinical outcome or focused instead on the steps taken during the diagnostic process was considered process related. Additionally, to assess the use of linguistic politeness strategies, the GP staff members scored whenever polite language was present, such as hedging (e.g., plausibility shields like “I believe,” “I think,” “could have,” “a little,” and “fairly,” as well as the use of question marks), impersonalization (e.g., omitting names or pronouns to create distance), and conventional indirectness (e.g., phrases like “good,” “solid,” or “met expectations”) based on the research of Ginsburg et al.^21^ After establishing consensus on coding rules, the 2 GP staff members collaboratively coded the feedback of 3 participants (multiplied by 6 cases per participant). After this, they independently coded the feedback of 10 more participants each (multiplied by 6 cases per participant). Interrater reliability analysis, using the *κ* statistic, indicated moderate to substantial agreement between the raters for these 10 participants (process: *κ* = 0.62; 95% confidence interval [CI], 0.54–0.70; *P* < .001; outcome: *κ* = 0.52; 95% CI, 0.21–0.83; *P* < .001; politeness strategy: *κ* = 0.74; 95% CI, 0.61–0.87; *P* < .001). After a second consensus meeting, the GPs each coded half of the remaining 49 participants’ feedback (see Supplemental Digital Appendix 2 at http://links.lww.com/ACADMED/B732 for an example of coded feedback).

#### Feedback quantity.

To evaluate feedback quantity, a repeated-measures ANOVA was conducted to analyze the mean total idea units provided in the feedback. Vignette version (adverse vs favorable clinical outcomes) and feedback valence (negative vs positive feedback) were used as within-subjects factors to assess the influence of supervisors’ knowledge of the clinical outcome of the case vignette and the valence of the feedback on the quantity of feedback provided.

#### Feedback characteristics.

Subsequently, the characteristics of the feedback were assessed by examining the effects of the clinical outcome of the vignette (adverse vs favorable) and valence of the feedback (negative vs positive) on the mean proportion of specific idea units (feedback specificity) and the mean proportion of process-focused idea units (feedback focus), using 2 separate repeated-measures ANOVAs. Again, vignette version and feedback valence were taken as within-subjects factors.

#### Politeness strategies.

Additionally, to analyze the use of polite language, the effects of clinical outcome of the vignette (adverse vs favorable) and valence of the feedback (negative vs positive) on the mean proportion of feedback that included a politeness strategy were examined. This analysis used a repeated-measures ANOVA, again with vignette version and feedback valence as within-subject factors.

## Results

### Participants

Sixty-three supervisors were asked to participate in the study. Of the 62 who agreed to participate, 57 were GPs, 2 were internists, 2 were geriatricians, and 1 was a dermatologist, all supervising GP residents in clinical practice in various settings depending on the stage of their training program (first and third years in GP; second year in diverse settings, such as elderly care, emergency care, or dermatology). Twenty-nine were supervising a first-year resident, 4 were supervising a second-year resident, 24 were supervising a third-year resident, and 5 were not currently supervising a resident. Twenty-six (41.9%) were male, and 36 (58.1%) were female. Mean (SD) age was 49.8 (8.3) years (range, 35–65 years). Mean (SD) length of experience was 19.5 (8.7) years (range, 4–40 years) as a medical specialist and 7.9 (6.4) years (range, 1–24 years) as a supervisor.

### Diagnostic performance scores

#### Main effects of the clinical outcome of the vignette and moment of scoring on diagnostic performance scores.

**Figure 2:**
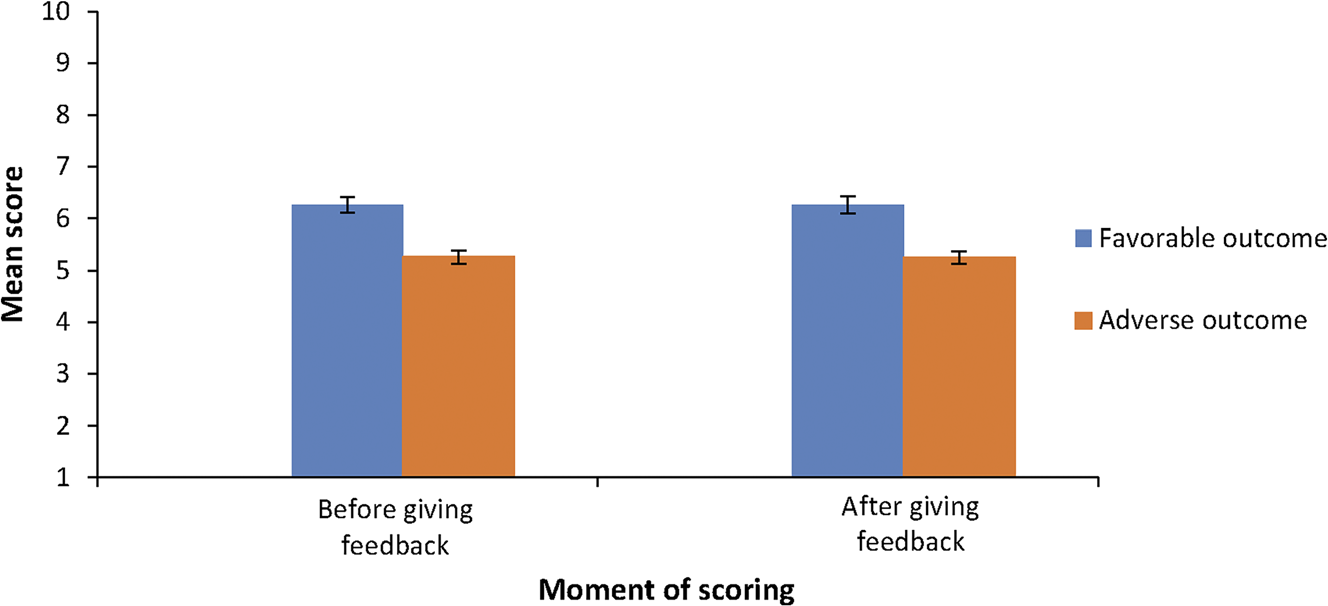
Mean diagnostic performance scores based on clinical outcome of the vignette and moment of scoring for 62 general practice supervisors assessing residents’ diagnostic performance in 6 fictitious, ambiguous clinical case vignettes with favorable or adverse clinical outcomes, Erasmus Medical Center, June 2023. Scores are measured on a scale of 1 to 10, with higher scores indicating better performance. Error bars indicate SEs.

Figure 2 shows the mean diagnostic performance scores before and after providing feedback for vignettes with adverse and favorable clinical outcomes. A significant difference was found based on the vignette version (*F*_1,61_ = 40.92, *P* < .001, *η*^2^ = 0.40), indicating that scores were generally lower for cases with adverse clinical outcomes compared with favorable clinical outcomes (mean [SE] scores, 5.25 [0.12] vs 6.26 [0.16]; *P* < .001). However, there was no significant difference based on the moment of scoring (before or after providing feedback), suggesting that reflecting on feedback did not change the scores (*F*_1,61_ = 0.04, *P* = .85, *η*^2^ = 0.001).

#### Interaction effect between the clinical outcome of the vignette and moment of scoring on diagnostic performance scores.

There was also no significant interaction between vignette version and the timing of the scoring (*F*_1,61_ = 0.004, *P* = .95, *η*^2^ = 0.00). This finding indicates that the difference in scores between adverse and favorable clinical outcomes was consistent before and after feedback, with no influence from reflection.

### Feedback quantity

#### Main effects of the clinical outcome of the vignette and feedback valence on feedback quantity.

**Figure 3:**
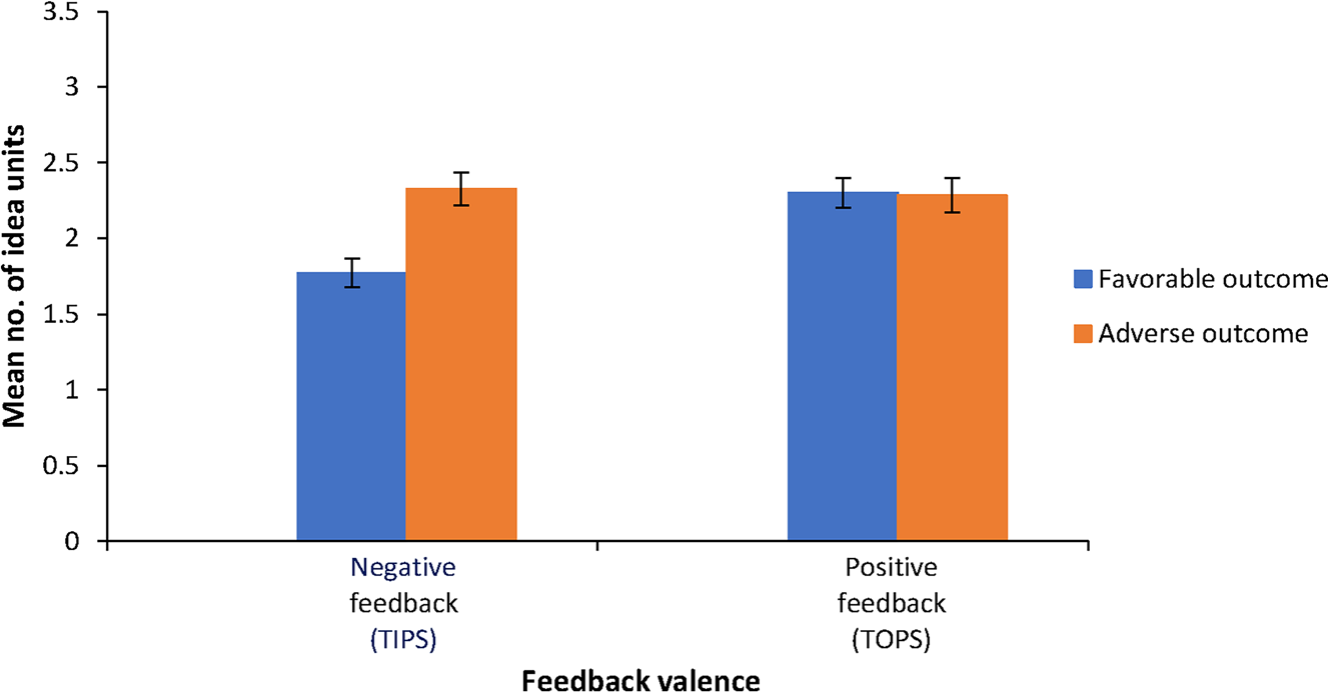
Mean number of idea units based on clinical outcome of the vignette and feedback valence for 62 general practice supervisors assessing residents’ diagnostic performance in 6 fictitious, ambiguous clinical case vignettes with favorable or adverse clinical outcomes, Erasmus Medical Center, June 2023. Error bars indicate SEs.

Figure 3 shows the mean number of idea units in the feedback for vignettes with adverse and favorable clinical outcomes as well as for positive and negative feedback (feedback valence). Both the clinical outcome of the vignette and the valence of the feedback significantly influenced the number of idea units in the feedback: adverse clinical outcomes resulted in more idea units than favorable clinical outcomes (2.31 vs 2.05, *F*_1,61_ = 13.14, *P* < .001, *η*^2^ = 0.18), and positive feedback contained more idea units than negative feedback (2.29 vs 2.07, *F*_1,61_ = 6.32, *P* = .02, *η*^2^ = 0.09).

#### Interaction effect between the clinical outcome of the vignette and feedback valence on feedback quantity.

There was also a significant interaction between the clinical outcome of the vignette and feedback valence (*F*_1,61_ = 16.16, *P* < .001, *η*^2^ = 0.21), meaning that the influence of knowledge of the clinical outcome of the vignette on the number of idea units differed for positive and negative feedback. Specifically, negative feedback (TIPS) showed significantly more idea units for adverse clinical outcomes than favorable ones (mean [SE], 2.35 [0.11] vs 1.80 [0.09]; *P* < .001). However, positive feedback (TOPS) showed a similar number of idea units across both clinical outcomes (mean [SD], 2.28 [0.12] vs 2.31 [0.10]; *P* = .78). For adverse clinical outcome vignettes, negative and positive feedback contained similar amounts of idea units (mean [SD], 2.35 [0.11] vs 2.28 [0.12]; *P* = .57), whereas for favorable clinical outcome vignettes, positive feedback had more idea units than negative feedback (mean [SE], 2.31 [0.10] vs 1.80 [0.09]; *P* < .001).

### Feedback characteristics

#### Main effects of the clinical outcome of the vignette and feedback valence on feedback specificity.

**Figure 4:**
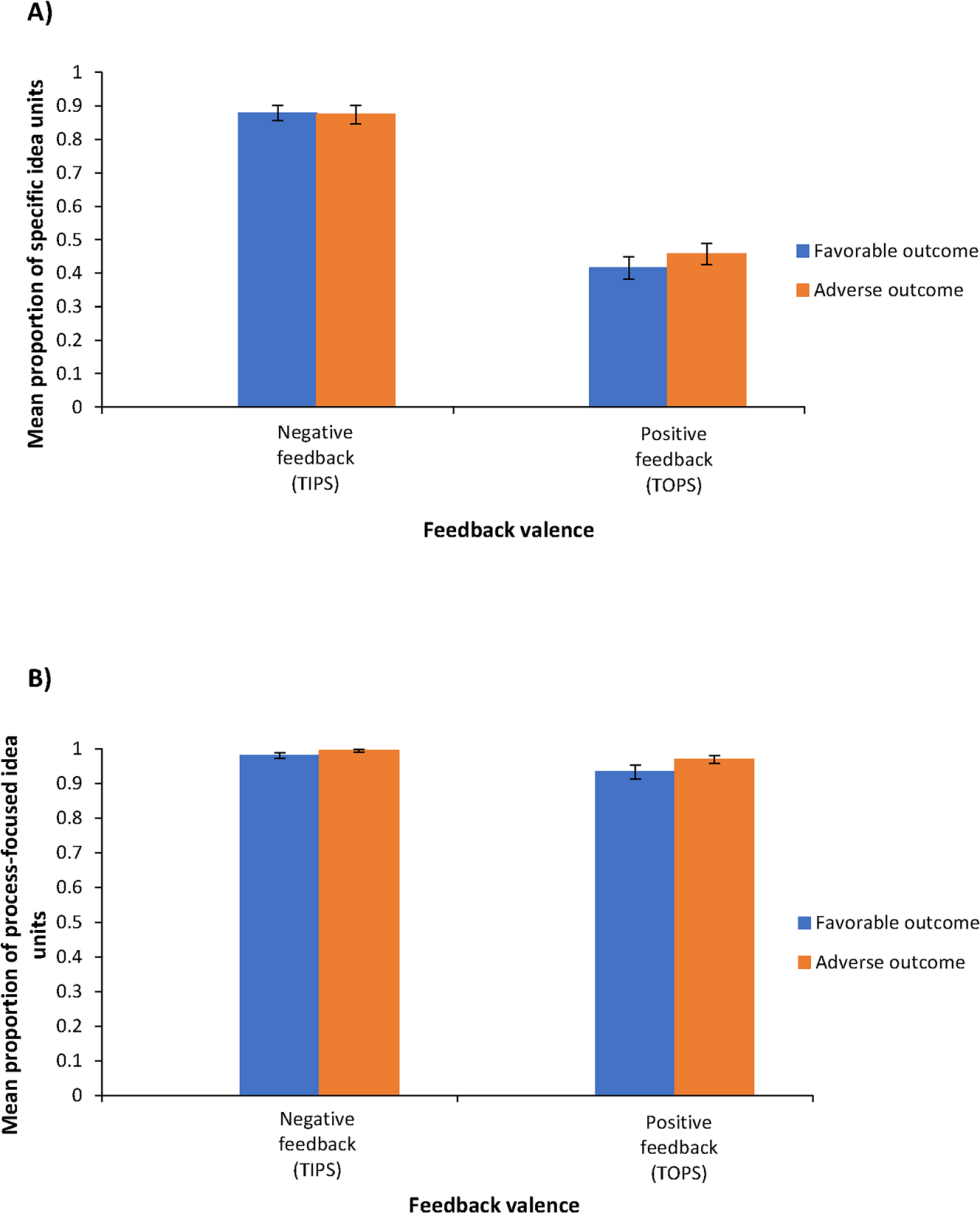
Specific and process-focused idea units. (A) Mean proportion of specific idea units based on clinical outcome of the vignette and feedback valence for 62 general practice supervisors assessing residents’ diagnostic performance in 6 fictitious, ambiguous clinical case vignettes with favorable or adverse clinical outcomes, Erasmus Medical Center, June 2023. Error bars indicate SEs. (B) Mean proportion of process-focused idea units based on clinical outcome of the vignette and feedback valence for 62 general practice supervisors assessing residents’ diagnostic performance in 6 fictitious, ambiguous clinical case vignettes with favorable or adverse clinical outcomes, Erasmus Medical Center, June 2023. Error bars indicate SEs.

Figure 4A displays the mean proportion of specific idea units in feedback for vignettes with adverse and favorable clinical outcomes and for positive and negative feedback (feedback valence). The clinical outcome of the vignette did not significantly affect the proportion of specific idea units (*F*_1,61_ = 0.70, *P* = .41, *η*^2^ = 0.01), meaning that knowing the clinical outcome of the vignette did not impact how specific the feedback was. However, the valence of the feedback had a significant effect on the specificity of the idea units (*F*_1,61_ = 191.36, *P* < .001, *η*^2^ = 0.76): negative feedback contained a statistically significant higher proportion of specific idea units than positive feedback (mean [SE], 0.88 [0.02] vs 0.44 [0.03]; *P* < .001).

#### Interaction effect between the clinical outcome of the vignette and feedback valence on feedback specificity.

There was no significant interaction between knowledge of the clinical outcome of the vignette and feedback valence (*F*_1,61_ = 0.95, *P* = .33, *η*^2^ = 0.02), indicating that positive and negative feedback had a similar effect on the specificity of the feedback regardless of whether the clinical outcome was favorable or adverse.

#### Main effects of the clinical outcome of the vignette and feedback valence on feedback focus.

Figure 4B shows the mean proportion of process-focused idea units in feedback for vignettes with adverse and favorable clinical outcomes as well as for positive and negative feedback (feedback valence). Nearly all feedback addressed process-related aspects (grand mean proportion of process-related idea units, 0.97; 95% CI, 0.95–0.98). Both vignette clinical outcome and feedback valence significantly affected the proportion of process-focused ideas (*F*_1,61_ = 8.48, *P* = .005, *η*^2^ = 0.12, and *F*_1,61_ = 5.51, *P* = .02, *η*^2^ = 0.08, respectively): adverse clinical outcomes had a higher proportion of process-focused feedback than favorable clinical outcomes (0.98 vs 0.96). Negative feedback also had a higher proportion of process-focused ideas compared with positive feedback (0.99 vs 0.95), with both differences small but statistically significant.

#### Interaction effect between the clinical outcome of the vignette and feedback valence on feedback focus.

There was no significant interaction between knowledge of the clinical outcome of the vignette and feedback valence (*F*_1,61_ = 1.43, *P* = .24, *η*^2^ = 0.02), indicating that positive and negative feedback had similar effect on the focus of feedback, regardless of whether the clinical outcome was favorable or adverse.

### Politeness strategies

#### Main effects of the clinical outcome of the vignette and feedback valence on presence of politeness strategies.

**Figure 5:**
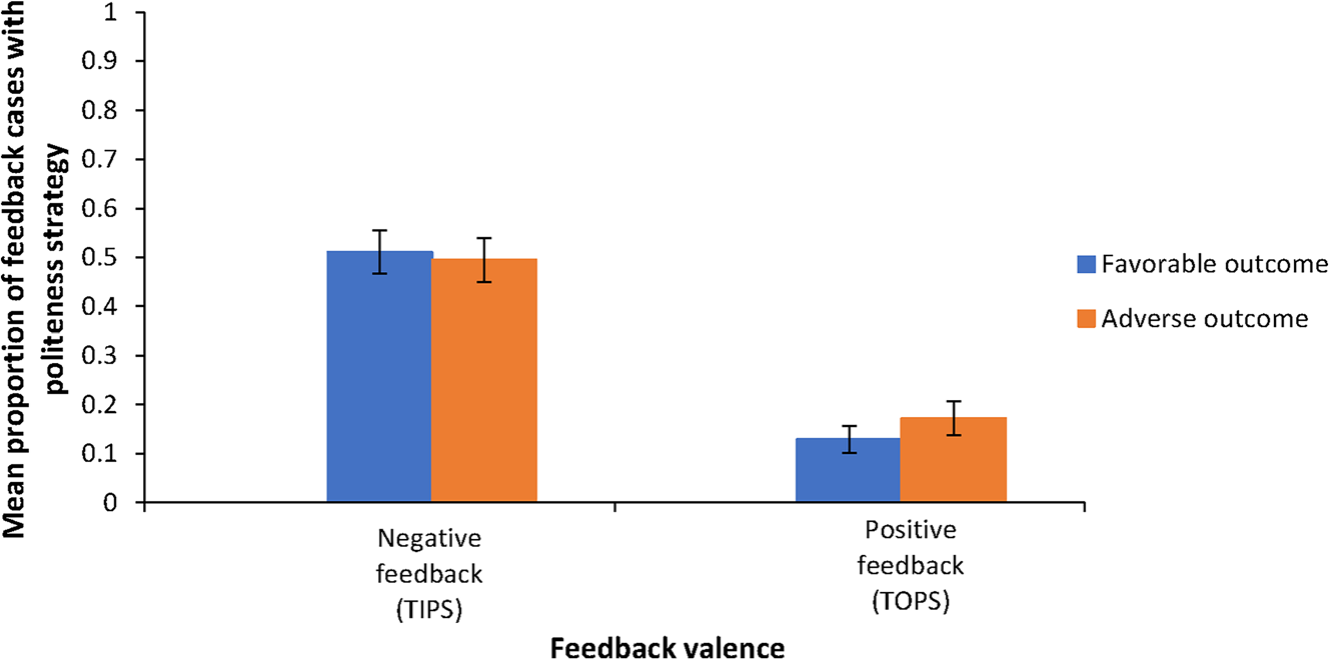
Mean proportion of feedback cases with a politeness strategy based on clinical outcome of the vignette and feedback valence for 62 general practice supervisors assessing residents’ diagnostic performance in 6 fictitious, ambiguous clinical case vignettes with favorable or adverse clinical outcomes, Erasmus Medical Center, June 2023. Error bars indicate SEs.

Figure 5 illustrates the mean proportion of feedback where politeness strategies were used in feedback for vignettes with adverse and favorable clinical outcomes as well as for positive and negative feedback (feedback valence). The clinical outcome of the vignette did not significantly affect the use of politeness strategies (*F*_1,61_ = 0.15, *P* = .70, *η*^2^ = 0.002), whereas feedback valence had a significant impact (*F*_1,61_ = 87.84, *P* < .001, *η*^2^ = 0.59). Specifically, the proportion of feedback using politeness strategies was higher in negative feedback than in positive feedback (mean [SE], 0.50 [0.04] vs 0.15 [0.02]; *P* < .001).

#### Interaction effect between the clinical outcome of the vignette and feedback valence on presence of politeness strategies.

There was no significant interaction between vignette version and feedback valence (*F*_1,61_ = 0.70, *P* = .41, *η*^2^ = 0.01), indicating that positive and negative feedback had a similar effect on the presence of politeness strategies, regardless of whether the clinical outcome was favorable or adverse.

## Discussion

This study demonstrates the presence of outcome bias in supervisors’ evaluation of CR performance among GP residents. Despite identical diagnostic processes in clinical vignettes with 2 possible diagnoses, cases with adverse clinical outcomes (inaccurate diagnoses) received significantly lower diagnostic performance scores—1 point lower on a 10-point scale—and feedback averaged 2.35 negative idea units (0.55 more than the 1.80 negative idea units for favorable outcomes), reflecting an estimated 31% increase in negative feedback for adverse outcomes. Negative feedback exhibited a significantly higher proportion of specific idea units (0.88) than positive feedback (0.44), regardless of the vignette’s clinical outcome. The proportion of idea units in all feedback addressing process-related aspects constituted a clear majority, with a grand mean of 0.97—regardless of vignette outcome or feedback valence. Additionally, polite language was more prevalent in negative feedback (proportion, 0.50) than in positive feedback (proportion, 0.15), independent of the vignette’s clinical outcome.

Although a score difference of 1 point may seem inconsequential, the difference between a score of 6.25 and 5.25 could mean the distinction between passing and failing. Considering that this judgment is assigned for the same description of diagnostic reasoning process and decisions, this scoring difference can only result from the assessors’ awareness of the clinical outcome of the case. Another factor to take into account when interpreting the scores is that the 10-point scale may not inherently signify a spectrum of 10 unique numbers. Although scores ranging from 1 to 10 are commonly used in the Dutch educational system, supervisors may, in practice, refrain from assigning extremely low or high scores. As a result, the used score ranges often fall between 4 and 9, further amplifying the importance of a 1-point difference.

Our data indicate that vignettes with adverse clinical outcomes lead to 31% more idea units in negative feedback (TIPS) compared with vignettes with favorable clinical outcomes. Conversely, there was no significant difference in the number of idea units in positive feedback (TOPS) between vignettes with adverse or favorable clinical outcomes. This finding resulted in a predominance of positive feedback in vignettes with favorable clinical outcomes, whereas vignettes with adverse clinical outcomes had nearly equal amounts of positive and negative feedback. This difference in feedback distribution could influence how residents perceive the feedback. When positive feedback outweighs negative feedback for favorable clinical outcomes, it might reinforce residents’ confidence and motivation, fostering a positive learning experience. However, increased negative feedback for adverse clinical outcomes, combined with lower scores, may be perceived as a threat to oneself, potentially impairing performance^16^ and obstructing learning by triggering negative emotions, such as insecurity or fear.

How individuals process feedback is significantly influenced by personal characteristics and circumstances, including their emotional state, experience level, and previous experiences with feedback. Additionally, the impact of feedback is affected by the receiver’s regulatory focus, as outlined in regulatory focus theory.^31^ In a prevention focus (concerned with punishments, such as diagnostic errors), negative feedback may enhance motivation and performance, whereas positive feedback may diminish it. Conversely, in a promotion focus (concerned with rewards and eagerness), negative feedback decreases motivation and performance, whereas positive feedback increases it.^12,16^ Therefore, in scenarios in which a prevention focus is relevant, such as dealing with diagnostic errors and adverse clinical outcomes, providing negative feedback may offer valuable learning opportunities for students, thus underscoring the value of using diagnostic errors with adverse clinical outcomes, such as malpractice cases, for educational purposes.

Another argument supporting the beneficial effect of negative feedback on learning is that negative feedback contained double the proportion of specific idea units (0.88) compared with positive feedback (0.44), irrespective of the vignette’s clinical outcome. Because content-specific feedback is generally considered more effective than general information in medical training, it has the potential to enhance motivation and performance.^30^ Therefore, providing more negative, yet content-specific feedback for cases with adverse clinical outcomes may offer more learning opportunities.

The finding that nearly all feedback (grand mean proportion, 0.97) addressed process-related aspects—regardless of vignette outcome or feedback valence—challenges the assumption that feedback for cases with adverse clinical outcomes primarily focuses on the missed diagnosis or outcome rather than the complexities of the diagnostic process itself. Although slightly more process-focused feedback was given for adverse clinical outcomes and negative feedback, the minimal differences from favorable clinical outcomes and positive feedback likely hold limited practical relevance for medical education. The predominance of feedback concentrating on specific aspects that went wrong in the reasoning process, even in cases with adverse clinical outcomes, suggests that diagnostic errors can indeed enrich the learning experience.^3^

Finally, supervisors used politeness strategies in half of all negative feedback instances (mean proportion, 0.50) compared with positive feedback (mean proportion. 0.15)—more than 3 times more often—suggesting a deliberate effort to soften the impact of corrective comments. This approach could help mitigate some of the adverse effects associated with the increased amount of negative feedback triggered by knowledge of the clinical outcome of a case. However, there is still room for improvement in leveraging polite language to enhance feedback delivery.

### Recommendations

Cautioning reviewers against letting knowledge of the clinical outcome of a case influence their judgment has proven ineffective.^1,3^ Complete withholding of the clinical outcome of the case until feedback is given is neither feasible nor desirable in daily medical practice because this study proves that clinical outcome knowledge enhances the provision of specific process-related feedback, thereby offering valuable additional learning opportunities. However, emphasizing that the narrative feedback might offer more learning opportunities than numerical scores and that separating the numerical score from the narrative feedback could enhance the effectiveness of the assessment process can be a valuable recommendation for supervisors assessing residents’ CR skills in daily practice. Establishing a trustworthy environment, especially in the case of a diagnostic error in which the feedback recipient is in a prevention-focused mindset, can facilitate learning from negative feedback.^19^ Use of linguistic politeness strategies, as GP supervisors commonly do when providing negative feedback, can contribute to creating such an environment. Moreover, supervisors should strive to enhance the specificity of feedback in all situations—both cases with favorable and those with adverse clinical outcomes and for both positive and negative feedback. This study revealed that, for positive feedback, supervisors tend to provide nearly equal proportions of specific and generic feedback. Particularly in situations with favorable clinical outcomes, there is potential for improvement by making positive feedback more specific because students who are in a promotional focus are most affected by positive feedback.

### Limitations

This study has several limitations. First, the use of written, fictitious, and ambiguous vignettes might not directly translate to real-life feedback scenarios in clinical practice. The nuances of how feedback is delivered and received, along with contextual factors in actual clinical settings, cannot be fully captured in written vignettes. In authentic clinical settings, feedback is delivered and received through nuanced verbal and nonverbal communication, shaped by the interpersonal dynamics between feedback givers and receivers. The static, written vignette format used in this study does not capture these complex, real-time settings and interactions, which may limit our understanding of how feedback might be internalized and acted on by residents in a real-world context. This study did not directly examine the impact of biased feedback on residents, which would provide further insights into how feedback with outcome bias affects learning and development.

Second, the study’s reliance on a quantitative approach to analyze feedback limits the ability to capture nuanced language and contextual elements. Although this quantitative study approach facilitated the identification of broad patterns related to outcome bias, a detailed qualitative analysis could provide further insight into the subtleties of bias manifestation.

Third, the controlled nature of this study does not capture the full complexity of clinical settings, especially regarding the intricacies of the diagnostic process. Real-world diagnostic errors are rarely simple and often involve multiple interacting factors. These complexities and their interplay were out of the scope of the current study and therefore not fully accounted for in this study. Future research could benefit from qualitative methods and a focus on these intricate interactions to deepen our understanding of the influence of outcome bias in feedback on resident performance.

Fourth, the research was performed within the GP department of a single academic university in the Netherlands, where the educational system dedicates time for CR assessments in practice and supervisors receive training in feedback delivery. This setting may limit the generalizability of the findings to locations in other parts of the world where there is less emphasis on CR skills and/or on supervisors’ training. Cultural variations, particularly in feedback provision, use of polite language, management of diagnostic errors, and associated emotions, were also not considered.

### Conclusions

This study demonstrates the presence of outcome bias in assessments of residents’ CR skills. Supervisors assigned lower diagnostic performance scores and provided more negative feedback when a vignette resulted in an adverse clinical outcome compared with when the same diagnostic process led to a favorable outcome. This negative feedback, characterized as specific and process related, may contribute to an enhanced learning experience by focusing on aspects that went wrong in the reasoning process. This finding underscores the educational potential of diagnostic errors, as discussed in previous studies.^8,9^ However, it is essential to acknowledge that these lower scores and negative feedback may potentially evoke negative emotions, which could hinder the learning and performance of residents. Our findings indicate that supervisors try to alleviate potential negative emotions triggered by negative feedback—emotions that may impede learning—by incorporating linguistic politeness strategies into their negative feedback. These results emphasize the need for strategies to assess CR skills in daily practice more objectively to maximize learning opportunities.
